# CEPP regimen (cyclophosphamide, etoposide, procarbazine and prednisone) as initial treatment for Hodgkin lymphoma patients presenting with severe abnormal liver function

**DOI:** 10.1186/2050-7771-2-12

**Published:** 2014-06-23

**Authors:** Keyur Thakar, Aileen Novero, Arundhati Das, Adriana Lisinschi, Bella Mehta, Tauseef Ahmed, Delong Liu

**Affiliations:** 1 Division of Hematology & Oncology, Department of Medicine, Westchester Medical Center, 100 Woods Rd, Valhalla, NY 10595, USA; 2 Henan Tumor Hospital, Zhengzhou University, Zhengzhou 450003, China

**Keywords:** CEPP regimen, Hodgkin lymphoma, extranodal lymphoma

## Abstract

ABVD regimen (doxorubicin, bleomycin, vinblastine and dacarbazine) remains the most commonly used front-line therapy for Hodgkin lymphoma. However, atypical and extranodal presentations present challenges to initial therapy, especially in the presence of renal and liver failure. We hereby present two cases of young male patients with atypical presentation of Hodgkin lymphoma with severe abnormal liver function. Patients showed excellent response to cyclophosphamide, etoposide, procarbazine and prednisone (CEPP regimen).

## Background

Hodgkin lymphoma (HL) is characterized by the presence of Reed-Sternberg cells and lymphadenopathy [1]. There are two histological types of HL: nodular lymphocyte predominant and classical HL (cHL). cHL has four entities: nodular sclerosis, mixed cellularity, lymphocyte depletion, and lymphocyte-rich. Most patients with HL present with asymptomatic superficial lymphadenopathy. Extranodal involvement is uncommon and seen only in 15% of the cases where it is usually in advanced stages. On the other hand, hematogenous spread is seen in 5-10% of the cases [2].

Since its first description by Thomas Hodgkin in 1832, the treatment of HL has significantly changed [3-8]. Hodgkin lymphoma is a highly curable disease in the current era. Chemotherapy with doxorubicin, bleomycin, vinblastine and dacarbazine (ABVD regimen) remains the mainstay of treatment [4,5,9,10]. As per American Cancer Society’s estimates for HL in the United States, the 5-year and 10-year survival rates reached 85% and 80%, respectively [11]. However atypical presentations continue to pose a challenge [2,12].

We report two cases of HL with severe abnormal hepatic function. Since liver toxicity is a major adverse effect for doxorubicin, doxorubicin- containing regimens are less desired for this group of patients [13,14]. The patients instead received cyclophosphamide, etoposide, procarbazine and prednisone (CEPP regimen) with remarkable response.

## Case presentation

### Case 1

A 38 year-old male with no significant past medical history presented with fever for two months. He was initially treated for bronchitis. Patient soon developed bilateral hearing loss and bilateral foot pain. He was started on doxycycline and prednisone by his local physician with no improvement. Patient had persistent fever and developed changes in his mental status. He was admitted to a local hospital and found to have pancytopenia and elevated transaminases. He was transferred to Westchester Medical Center for further management. Initial laboratory data showed acute liver injury (AST: 368 IU/L, ALT: 486 IU/L, alkaline phosphatase: 183 IU/L, total bilirubin: 14.6 mg/dL, direct bilirubin: 10.2 mg/dL) and pancytopenia (WBC: 1500/mm3, Hemoglobin: 9.4 g/dL, Platelets: 59,000/dL). Serologies for hepatitis, Lyme disease, Ehrlichiosis, Babesiosis, and Rickettsia were all negative. Cerebrospinal fluid did not reveal anything significant. MRI of feet revealed myofascitis and cellulitis and that of left foot showed myositis. CT scan of the chest, abdomen and pelvis revealed no lymphadenopathy. Patient then underwent a liver biopsy. The liver tissue stained positive for Epstein-Barr virus (EBV) on immunohistochemistry. The serum EBV DNA level was 49,054 IU/dL. The bone marrow biopsy at that time revealed severely myelodepleted marrow core with marked reticulin fibrosis and negative for EBV. Flow cytometry analysis revealed a mixed population of cells with polytypic B cells positive for CD20, but no evidence of lymphoma.

Patient was started on cidofovir, an anti-viral medication, which had to be discontinued later due to worsening renal failure. Since his symptoms were thought to be possibly due to a lymphoproliferative disease from EBV and that flow cytometry was positive for CD20, a trial of rituximab 375 mg/m2 for one dose was given. After this single dose of rituximab, his liver enzymes and blood counts significantly improved. He was subsequently discharged to home. His laboratory data upon discharge were as follows: WBC 2100/mm3, Hemoglobin 8.1 g/dL, Platelets 329,000/dL, BUN 15 mg/dl, creatinine 1.17 mg/dl, AST 44 IU/L, ALT 87 IU/L, alkaline phosphatase: 345 IU/L, total bilirubin: 2.3 mg/dL, direct bilirubin 1.7 mg/dL.

Two months later, he again presented with altered mental status and was found to have elevated transaminases, persistent fever, pancytopenia and acute kidney injury (BUN: 136 mg/dL, Cr: 12.6 mg/dL). He was started on continuous veno-venous hemodialysis (CVVHD) and then transferred to our institution for further management.

Patient had persistent fever spikes despite being on broad-spectrum antibiotics. Pertinent laboratory data during this second admission revealed WBC: 3100/mm3, platelets: 29,000/dL, Hgb: 9.0 g/dL, AST: 392 IU/dL, ALT: 312 IU/dL, alkaline Phosphatase: 181 IU/dL, total bilirubin: 13 mg/dL, direct bilirubin: 10.3 mg/dL, lactate dehydrogenase: 819 IU/dL, fibrinogen: 141 mg/dL, uric acid: 8.7 mg/dL, BUN: 86 mg/dL and Cr. 7.9 mg/dL, HIV status non-reactive and urine myoglobin: 4064 μg/L, EBV DNA level was 112,073 IU/ml. Repeat bone marrow biopsy revealed classical Hodgkin lymphoma that was EBV positive with background trilineage hematopoiesis and occasional hemophagocytosis. The lymphoma cells have abundant cytoplasm and mono-, bi- or multinucleated irregular nuclear contour, clumped or smudged chromatin and prominent eosinophilic macronucleoli that are consistent with Reed-Sternberg cells. Immunohistochemical stains revealed large atypical cells uniformly positive for CD30 with typical membranous and dot patterns with focal variable positivity for CD15. The cells were negative for CD20, CD45, CD3, and ALK1. The atypical cells were also positive for EBV LMP-1. Thus, the diagnosis of classical Hodgkin lymphoma, EBV positive, was made.

MRI of the abdomen showed several T2 hyperintense lesions scattered throughout the liver and spleen, consistent with an infiltrative process. Liver biopsy was not done this time due to low platelet counts.

Given his deranged liver and renal function, ABVD regimen was not the best choice. Instead, the CEPP regimen (cyclophosphamide 600 mg/m2 in 250 ml NS on day 1 and 8; etoposide 70 mg/m2 in 500 ml NS on Days 1,2,3; procarbazine 60 mg/m2 PO daily on days 1–10, prednisone 60 mg/m2 daily PO on days 1–10) was used. The dosages of cyclophosphamide and etoposide were not reduced for the abnormal liver and renal functions since the patient was on dialysis, and the dosages of the two agents were not very high. Patient showed dramatic response and became afebrile on day 2. His renal and liver function tests continued to improve and he was taken off hemodialysis. His total bilirubin came down from 17 mg/dL before chemotherapy to 2.5 mg/dL upon discharge. Patient was later discharged to follow with his local oncologist for further therapy.

### Case 2

A 49 year-old male with history of HIV was referred to Westchester Medical Center for evaluation of persistent fever and abnormal liver function tests (LFT). He presented with complaints of recurrent episodes of high fevers, chills, night sweats, fatigue, itchiness for about 3 months. He also reported decreased appetite and weight loss of 25 lb. in the past year. Patient was seen as an outpatient by his Infectious Disease doctor and was found to have transaminitis and elevated alkaline phosphatase. Initially, it was thought that deranged hepatic tests were induced by Stribild, a combination pill of elvitegravir, cobicistat, emtricitabine, and tenofovir disoproxil fumarate for antiretroviral therapy. Stribild was thus discontinued. However, his abnormal liver function, fever, and weight loss were not improving and patient was referred for further evaluation. Clinically, the patient had no palpable lymphadenopathy nor hepatosplenomegaly. Initial laboratory data showed acute liver injury (AST: 359 IU/L, ALT: 437 IU/L, alkaline phosphatase: 2137 IU/L, total bilirubin: 2.5 mg/dL). A CBC showed WBC: 3500/mm3, Hemoglobin: 12.8 g/dL, Platelets: 339,000/dL. EBV DNA level was 1611 copies/ml. HIV status: CD 4 count 136 cells/uL, HIV –1 RNA level was <0 copies/ml and <1.30 Log copies/ml. Hepatitis A, B, C, and CMV as well as autoimmune panel for anti-mitocondrial and anti-smooth muscle antibodies were all negative.

CT scan of the chest, abdomen and pelvis revealed a normal-sized liver, mild splenomegaly of 14.7 cm, and retroperitoneal lymphadenopathy (LN) with the largest LN measuring 2.5 cm.

Patient then underwent a liver biopsy which was negative for infiltrative disease or granulomas. Immunostaining for EBV and HSV- 1/2 showed negative results. Due to scattered apoptosis and bile stasis related damages, suspicion for virus related pathology and/or drug related injury was raised, with no overt evidence of lymphoma. His laboratory data upon discharge was as follows: AST 302 IU/L, ALT 391 IU/L, alkaline phosphatase: 1871 IU/L, total bilirubin: 2.1 mg/dL, direct bilirubin 1.5 mg/dL.

On subsequent outpatient follow-up, he was found to have worsening anemia, LFT, and persistent fever. The patient was admitted to hospital again for re-evaluation. Pertinent laboratory data during this admission revealed WBC: 3700/mm3, Hgb: 7.7 g/dL platelets: 226,000/dL, AST: 99 IU/L, ALT: 94 IU/L, alkaline phosphatase: 1558 IU/L, total bilirubin: 5.5 mg/dL, direct bilirubin: 4.1 mg/dL, LDH: 271 IU/L, Na :129 mEq/L, BUN/Cr: 11 mg/dL and 0.61 mg/dL, HIV status: CD4 count 57 cells/uL, HIV –1 RNA level <20 copies/ml and <1.30 Log copies/ml and EBV DNA level was 5545 copies /ml. All blood cultures including bacterial, fungal and AFB as well as serological investigations for fungal and viral (with exception of EBV) panels were negative. Patient continued to be febrile despite administration of broad spectrum antibiotics.

A bone marrow biopsy was done. Pathology showed large atypical cells. Occasional large bi-nucleated cells with abundant amphophilic cytoplasm, prominent large nucleoli consistent with classic Reed-Sternberg cells were seen. Immunohistochemical stains showed that the large atypical cells were positive for CD30 and EBV, with rare cells positive for CD15. The large cells were negative for CD2, CD3, CD20 and CD45. In summary, the morphologic and immunophenotypic findings were consistent with involvement of the marrow by classical Hodgkin lymphoma.

Pathology from a repeat liver biopsy revealed foci of large atypical cells with large, mono or bi-lobated nuclei with abundant amount of cytoplasm, irregular nuclear contour, and prominent large nucleoli. The surrounding liver parenchyma was unremarkable. Immunohistochemical study showed that the large atypical cells were positive for CD30 and EBV, but negative for CD45, CD15, CD3 and CD20. CMV, Gram stains, PAS and AFB stains were negative. Taken together, the findings were suggestive of involvement of liver by Hodgkin lymphoma.

This patient was diagnosed to have classical Hodgkin lymphoma, stage IVb.

A CT scan of the chest, abdomen and pelvis showed interval increase in the size of liver (up to 24 cm) and spleen (up to 16 cm) with no focal hepatic or splenic lesion. Stable mediastinal, retroperitoneal and external iliac chain lymphadenopathy was seen.

Given his markedly abnormal liver function, the ABVD regimen was deemed not to be the best choice. Instead, the CEPP regimen was administered.

Patient showed a rapid response and became afebrile on day 2 of chemotherapy. His LFTs continued to improve. His total bilirubin of 5.5 mg/dL and alkaline phosphatase of 1558 U/L before chemotherapy were down to 2.2 mg/dL and 835 U/L respectively upon discharge on day 10 of the course. On outpatient follow-up, the LFTs continued to improve, and became virtually normal (AST: 35 IU/L, ALT: 52 IU/L, alkaline phosphatase: 394 IU/L, total bilirubin: 0.5 mg/dL). The patient had no further fever, and regained weight. The patient became negative on PET/CT scan. He appeared to have achieved unconfirmed complete remission. He has returned to work and started ABVD for further treatment. He remained in clinical remission and was completing the course of ABVD therapy at the last follow-up.

## Discussion

In comparison with extranodal non-Hodgkin’s lymphoma, extranodal HL is uncommon [2]. There are very few case reports for acute liver failure at the initial presentation [15-19] (Table 1). In one large series of 421 consecutive patients with HL, only six patients (1.4%) had liver abnormalities and no palpable lymphadenopathy. The diagnosis was made through liver biopsy [16]. In 2010, a case of a 20 year-old boy was reported to have similar presentation. However, he was managed only with intravenous antibiotics and he died within 25-days of admission [19]. In another case report from Chile, a 64 year-old lady presented with jaundice and malaise. She was diagnosed to have HL, mixed cellularity type. ABVD was avoided because of her hepatic involvement. Gemcitabine, cisplatin and dexamethasone were given with favorable results [20]. There was another case of a 50 year-old man presented with primary bowel involvement of HL with cholestatic liver function abnormality and renal failure. He was managed surgically followed by chemotherapy. They used nitrogen mustard and prednisone with the omission of vincristine and procarbazine. However the patient succumbed to pneumonia and other complications [18].

**Table 1:** Atypical Hodgkin’s Lymphoma with liver involvement

**Author**	**No. of cases**	**Clinical presentation**	**Hepatic functions**	**Regimen**	**Reference**
Thakar, et al., 2014	2	Case1: Fever, jaundice, altered mental status, renal failure, pancytopenia	Case1:	CEPP: Cyclophosphamide, Etoposide, Procarbazine, Prednisone	This paper
		Case 2: Fever, chills, night sweats fatigue, itchiness, weight loss	T Bil: 13 mg/dL		
			D Bil: 10.3 mg/dL		
			AST: 392 IU/dL ALT: 312 IU/dL		
			T Bil: 2.5 mg/dL		
			AST: 359 IU/dL ALT: 437 IU/dL		
Guha, et al., 2010	1	Fever, jaundice, weight loss, acute hepatic failure, enlarged lymph nodes	T Bil: 9 mg/dL	Only intravenous antibiotics and supportive care	[19]
			D Bil: 6 mg/dL		
			AST: 50 U/L		
			ALT: 80 U/L		
Orellana, et al., 2012	1	Jaundice, choluria, malaise, enlarged groin lymph nodes, and weight loss	T Bil: 18 mg/dL	Dexamethasone, Gemcitabine & Cisplatin (GDP) × 4 cycles followed by Adriamycin, Bleomycin, Vinblastine & Dacarbazine	[20]
			AST: 286 U/L		
			ALT: 177 U/L		
Rosque, et al., 2011	1	Abdominal pain, fever, jaundice, intermittent bloody diarrhea, weight loss, hepatosplenomegaly, bowel obstruction	T Bil: 10 mg/dL	High dose intravenous steroids, modified MOPP (mechlorethamine and prednisone without Vincristine & Procarbazine) × 3 cycles	[18]
			D bil: 7.8 mg/dL AST: 137 U/L		
			ALT: 199 U/L		

*T Bil* total bilirubin, *D Bil* direct bilirubin, *AST* aspartate aminotransferase, *ALT* alanine aminotransferase.

The current cases had severe abnormal liver function and unusual clinical courses. The diagnosis of cHL was confirmed for both patients on second hospital admission. EBV infection in HL is common [21-25]. Hepatic involvement is seen at presentation in 6-20% of the HL patients. The liver involvement is usually diffuse or infiltrative with patchy, irregular infiltrates originating in the portal areas. Therapy options and clinical data are very limited for HL in this type of patients [7,13,14,26,27]. As most of the conventional chemotherapy regimens could not be used in these cases, we used CEPP regimen based on prior experience with non-Hodgkin lymphoma (NHL) [28]. To our knowledge, this is the first report on the use of CEPP for initial treatment of HL.

## Conclusion

In conclusion, CEPP should be favored as an alternative regimen for selected HL patients who can not receive frontline treatment options like ABVD or other doxorubicin-containing regimen. It is unclear at this time whether patients should receive more cycles of CEPP or switch to ABVD regimen as further therapy. Brentuximab is used for CD30+ HL [3]. Novel agents should be studied for patients with Hodgkin lymphoma [29-34]. Clearly, more data are needed for the treatment of atypical cases of HL in this report.

## Consent

Informed consent for publication was obtained from the patients for both cases.

## Competing interest

The authors declare that they have no competing interests.

## Authors’ contributions

All authors contributed to the data collection, manuscript preparation and revisions. All authors have read and approved the final manuscript.
